# ER Stress and the UPR in Shaping Intestinal Tissue Homeostasis and Immunity

**DOI:** 10.3389/fimmu.2019.02825

**Published:** 2019-12-04

**Authors:** Olivia I. Coleman, Dirk Haller

**Affiliations:** ^1^Chair of Nutrition and Immunology, Technical University of Munich, Munich, Germany; ^2^ZIEL – Institute for Food & Health, Technical University of Munich, Munich, Germany

**Keywords:** UPR, immunity, tissue homeostasis, IBD, CRC

## Abstract

An imbalance in the correct protein folding milieu of the endoplasmic reticulum (ER) can cause ER stress, which leads to the activation of the unfolded protein response (UPR). The UPR constitutes a highly conserved and intricately regulated group of pathways that serve to restore ER homeostasis through adaptation or apoptosis. Numerous studies over the last decade have shown that the UPR plays a critical role in shaping immunity and inflammation, resulting in the recognition of the UPR as a key player in pathological processes including complex inflammatory, autoimmune and neoplastic diseases. The intestinal epithelium, with its many highly secretory cells, forms an important barrier and messenger between the luminal environment and the host immune system. It is not surprising, that numerous studies have associated ER stress and the UPR with intestinal diseases such as inflammatory bowel disease (IBD) and colorectal cancer (CRC). In this review, we discuss our current understanding of the roles of ER stress and the UPR in shaping immune responses and maintaining tissue homeostasis. Furthermore, the role played by the UPR in disease, with emphasis on IBD and CRC, is described here. As a key player in immunity and inflammation, the UPR has been increasingly recognized as an important pharmacological target in the development of therapeutic strategies for immune-mediated pathologies. We summarize available strategies targeting the UPR and their therapeutic implications. Understanding the balance between homeostasis and pathophysiology, as well as means of manipulating this balance, provides an important avenue for future research.

## The Endoplasmic Reticulum and Its Intricately Regulated Unfolded Protein Response

In mammalian cells, the extensive tubular-reticular network known as the endoplasmic reticulum (ER) forms a crucial site for maintaining calcium homeostasis, cholesterol production and lipid synthesis, and most importantly acts as a gatekeeper for synthesizing and folding secreted and transmembrane proteins (1). ER protein-folding can be disrupted by envrionmental, physiological and pathological factors, resulting in ER stress. Changes in calcium homeostasis, an altered redox status, energy deficiency, lipid overload or the accumulation of unfolded or misfolded proteins are examples of conditions that can disrupt the ER protein-folding environment (2, 3). Furthermore, perturbations in membrane fluidity through cellular lipid composition can cause lipotoxic ER stress (4, 5). The correct folding and post-translational modifications of proteins are necessary in order to maintain proteostasis within a cell, making it essential for the ER to have a rigorous quality control system should the ER environment become compromised. To this end, ER-associated degradation (ERAD) removes and subsequently degrades unfolded or misfolded proteins (6), and the ER unfolded protein response (UPR) serves to restore normal functioning of the cell (adaptation) or gears toward cell death (apoptosis) in case of irreversible disruption (2).

The UPR forms a conserved group of intracellular signaling pathways that primarily aim to restore ER homeostasis in response to ER stress caused by the accumulation of unfolded or misfolded proteins (7). The UPR consist of three membrane-bound signal transducers, namely PKR-like ER kinase (PERK), inositol-requiring enzyme 1 (IRE1) and Activating transcription factor 6 (ATF6) (8, 9). Under conditions of homeostasis, the luminal domains of these three signal transducers are bound to the chaperone Glucose regulated protein 78 (Grp78; also known as BiP and HSPA5) (10). Upon ER stress, Grp78 is translocated to the unfolded or misfolded proteins in the ER and thereby allows activation of the UPR signal transducers and subsequent downstream signaling (2, 11). Dissociation of Grp78 from the type-I transmembrane protein PERK activates its oligomerization and autophosphorylation in the cytosolic kinase domain, to allow PERK to phosphorylate the alpha subunit of translation initiation factor 2 (eIF2α) to indirectly inactivate the latter and inhibit mRNA translation (2). This leads to the inhibition of global protein synthesis, but specifically favors translation of mRNAs with short open reading frames such asactivating transcription factor 4 (ATF4), whose translation is induced (12). IRE1 is also a type-I transmembrane protein, which oligomerizes and subsequently autophosphorylates in the kinase domain to trigger its ribonuclease activity. This leads to unconventional splicing of Xbox-binding protein 1 (Xbp1) that binds to the UPR element (UPRE) to induce the transcription of UPR-related genes (13, 14). Upon release from Grp78, the type-II transmembrane protein ATF6 is transported to the Golgi where it is proteolytically cleaved via the site 1 and site 2 proteases (S1P and S2P) (15–17). Subsequently, the amino-terminal of ATF6 (nATF6) is translocated to the nucleus to bind to the ER stress response element (ERSE) for the transcription of genes encoding XBP1, ERAD components, and ER chaperones (13, 14, 18). Mechanistically, and unlike PERK and IRE1α, ATF6 is not dimerized and may perhaps be less affected by ER membrane changes such as reduced membrane fluidity.

In addition to the UPR being a response system that restores proteostasis, it fulfills an important signaling function that plays an anticipatory role in cells that have a higher protein folding demand and require an increased protein-folding capacity, in the absence of ER stress (12). Triggers that can activate this signaling response of the UPR include differentiating cells, such as maturing immune cells (19, 20), and hormones, such as epidermal groth factor (EGF) and vascular endothelial growth factor (VEGF) (21, 22). Thus, while the primary purpose of the UPR is to enhance protein degradation, reduce protein synthesis, increase ER protein folding capacity and upregulate chaperones required for protein folding, it has become increasingly clear that the UPR plays a crucial role in tissue homeostasis. The context-dependent functionality of the UPR, which tailors the output according to the cellular stimulus, render it an important gatekeeper of cellular physiology.

ER stress occurs under both physiological and pathological conditions, and has been associated with immune and inflammatory diseases (23), viral infection (24), cardiovascular diseases (25), diabetes (26), cancer (27), cerebral ischemia (28), neurodegenerative diseases (29), as well as mental disorders (30, 31). What remains to be elucidated, however, is whether the UPR plays a causal role in these pathologies or merely presents a consequence of the respective disease. Experimental models of the UPR provide a useful tool for unraveling the timely and mechanistic involvement of the UPR in disease development of the host. Indeed, to date there are a number of UPR-related mouse models that display disease phenotypes and provide insights into the role of this complex signaling pathway in physiology and pathology. Table 1 provides an overview of UPR-related mouse models and their associated disease phenotypes. From this group of mouse models, with targeted UPR pathway proteins, it is evident that the UPR plays a critical role in a diverse range of pathologies. Findings from such mouse models will be discussed in more detail in the following sections. In light of the complexity of the canonical UPR and its regulation, many of the *in vitro* cell culture studies on ER stress are difficult to translate into the *in vivo* situation. UPR-related mouse models are therefore indispensable to gain mechanistic insights into the role of the UPR in human disease.

**Table 1:** UPR-related mouse models and their associated disease phenotypes.

**UPR mouse model**	**Phenotype**	**References**
*Perk^−/−^*	Type I diabetes, bone abnormalities (early onset death)	(32)
*Ire1α^−/−^*	Embryonically lethal due to liver hypoplasia; liver deletion: hypolipidemia	(33, 34)
*nATF6^*IECtg*/*tg*^*	Colonic adenomas	(35)
*Atf6α^−/−^; p58^−/−^*	Embryonically lethal	(36)
*Atf6α^−/−^; Atf6β^−/−^*	Embryonically lethal	(37)
*Xbp1^−/−^*	Embryonically lethal due to liver hypoplasia; liver deletion: hypolipidemia; IEC deletion: enteritis; pancreatic acinar cell deletion: extensive pancreas regeneration; pancreatic β cell deletion; hyperglycemia.	(38–40)
*XBP1^*flox*/*flox*^VCre (XBP1^−/−^)*	Spontaneous intestinal inflammation	(39)
*Xbp1^+/−^*	Insulin resistance and type II diabetes on high fat diet	(38, 40)
*eIF2αS51A*	Perinatal death with diabetes and pancreatic β cell deficiency	(41)
*wfs1-mutant*	Diabetes and growth retardation	(42)
*CHOP^−/−^*	Protected from induced ER stress and type II diabetes	(43, 44)
*Chop^*IECTg*/*Tg*^*	Imparied mucosal repair	(45)
*Grp78*	Emryonically lethal; liver deletion: liver damage and hepatic steatosis	(46, 47)
*Grp94*	Embyonically lethal; B cell deletion: reduced antibody production; Bone marrow deletion: haematopoietic stem cell expansion	(48)
*Atf4^−/−^*	Embrionically or preinatally lethal	(49)
*Nrf2^−/−^*	Regenerative immune-mediated hemolytic anemia	(50, 51)
*P58^*IPK*−/−^*	Type I diabetes	(52)
*Cnx^−/−^*	Postnatal death	(53)
*Crt^−/−^*	Embrionically lethal	(54)
*Casp12^−/−^*	Resistant to ER-stress induced apoptosis	(55)
*Agr2^−/−^*	Terminal ileitis and colitis	(56)

## UPR in Immune Cells and Immune Barrier Function

**Figure 1:**
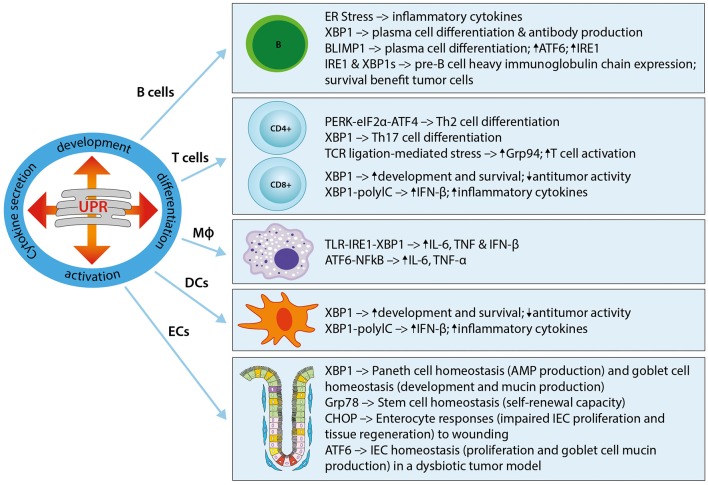
The UPR in immune cells and immune barrier function. The UPR plays a critical role in the development, differentiation, activation, and cytokine secretion of immune cells. Shown here are the effects of different UPR components on the main immune cell types, namely B cells, T cells (CD4+ and CD8+), macrophages (MΦ), and dendritic cells (DCs), as well as epithelial cells (ECs). Th2, T helper type 2; Th17, T helper type 17; Grp94, Glucose regulated protein 94; IL-6, Interleukin-6; IFN-β, Interferon-β; TNF, Tumor necrosis factor; TLR, Toll-like receptor; NFκB, Nuclear factor “kappa-light-chain-enhancer” of activated B-cells; ECs, epithelial cells; IEC, intestinal epithelial cell; AMP, antimicrobial peptide.

The UPR plays a critical role in the development of immune cells, with numerous studies highlighting its involvement in physiological immune processes (57). Discussed here, and summarized in Figure 1, is our understanding of the roles of ER stress and the UPR in immune responses that lead to immune activation, differentiation, and cytokine expression in immune cells. The UPR regulates cytokine production on multiple levels, extending from pattern recognition receptor (PRR) sensing to inflammatory signaling and cytokine transcription factor activation. UPR-PRR synergy that strengthens the immune response has been described by several groups and summarized in Claudio et al. (58) and Smith (59).

### UPR in Immune Cells

An essential role for the UPR in **B cells** was first described in studies that showed UPR activation in the differentiation of B cells into plasma cells, and a requirement for the induction of *Xbp1* for this process (38, 60–63). Further work revealed that the induction of XBP1is a differentiation-dependent event rather a response to increased immunoglobulin secretion (19, 20). XBP1 induces ER expansion in plasma cells, allowing for high immunoglobulin synthesis, and its deficiency abrogates immunoglobulin secretion by activated B cells through IRE1α hyperactivation (60, 62, 64, 65). Plasma cell differentiation is in part regulated by the transcription factor B lymphocyte-induced maturation protein 1 (Blimp-1) (62). Blimp-1 deficient B cells cannot activate transcription of plasma cell-related genes, including XBP1 (62). XBP1 is downstream of Blimp-1, as demonstrated in XBP1-deficient mice that resulted in normal Blimp-1 induction (62). Furthermore, Blimp-1 was shown to transcriptionally regulate ATF6 and IRE1 (66). XBP1 and also IRE1 are required during the pre-B cell stage during which immunoglobulin heavy chains are expressed for the first time, and XBP1 provides a survival benefit for tumor cells in pre-B acute lymphoblastic leukemia (33, 67).

In **T cells**, the UPR seems to play a role during cell differentiation. For example, the PERK-eIF2α-ATF4 axis has been implicated in Th2 cell differentiation, resulting in the upregulation of UPR genes (68). Similarly, XBP1 was shown to play a role in Th17 cell differentiation in response to inflammatory and autoimmune diseases (69, 70). Evidence for the important role played by the ER stress response in T cell activation was recently shown in a study where the ER molecular chaperone Grp94 was induced in CD4+ T cells following T cell receptor-ligation mediated ER stress (71). In turn, Grp94 deletion resulted in an activation defect. In CD8+ T cells, the IRE1α-XBP1 pathway activated upon acute infection was shown to be vital for effector T cell differentiation through increased expression of killer cell lectin-like receptor G1 (KLGR1) (72).

Both the development and the survival of antigen-presenting **Dendritic cells** (DCs) is driven by XBP1, with XBP1-deficiency resulting in reduced numbers of conventional and plasmacytoid DCs and increased apoptosis (73, 74). Interestingly, a recent study could also show a role for XBP1 in the suppression of antitumor immunity through the promotion of lipid accumulation and impaired antigen presentation (75). Further evidence for an important role of ER stress in DCs is shown by its ability to induce IFN-β production and IL-23 expression (74, 76). In DCs stimulated with the toll-like receptor (TLR) agonist polyinosinic:polycytidylic acid (PolyIC), silencing of XBP1 was shown to inhibit IFN-β production, whereas overexpression of XBP1 augmented inflammatory responses (74). TLR agonist stimulation of DCs under ER stress enhanced IL-23p19 expression, a target of the ER stress-induced transcription factor C/EBP homologous protein (CHOP), by stimulating the enhanced binding of CHOP to its promoter (76). In line with this, knockdown of CHOP reduced the expression of IL-23 *in vitro* (76). In phagocytic **macrophages**, the IRE1α-XBP1 ER stress axis is crucially involved in macrophage cytokine (IL-6, TNF, and IFN-β) responses to toll-like receptor (TLR) ligation in a pathway that involves TNF receptor-associated factor 6 (TRAF6) and the NAPDH oxidase-2 (NOX2) (77, 78). Furthermore, the IRE1α-XBP1 axis has also been implicated in the regulation of inflammatory cytokine (IL1-β) production via the activation of glycogen synthase kinase 3β (GSK3β) (79). A role for ATF6 in macrophages was demonstrated in a study of liver ischemia perfusion injury by Rao et al. in which prolonged ischemia activated the ATF6 arm of the UPR and subsequent pro-inflammatory cytokine production (TNF-α and IL-6) (80). It is important to note that the context-specific response and control of the individual UPR pathways is vital for the required immune response. For example, the survival of macrophages during an immune response is facilitated through the suppression of CHOP, downstream of ATF4 in the PERK pathway of the ER stress response (77, 81).

### UPR in Immune Barrier Function

In addition to the traditionally classified immune cells described above, **epithelial cells** lining mucosal surfaces, such as intestinal, gastric, and pulmonary surfaces, are further regulators of innate and adaptive immune responses. As the largest barrier between the host and the external environment, the gastrointestinal tract, with its enteroendocrine, absorptive, Paneth cells, and goblet cells, is particularly dependent on correct cellular functioning to maintain a state of intestinal homeostasis. Mucus-producing **goblet cells**, immunoglobulin-, chemokine-, and cytokine-secreting absorptive **enterocytes**, as well as anti-microbial peptide-producing **Paneth cells** have been shown to be particularly dependent on the UPR. For example, specific deletion of XBP1 in intestinal epithelial cells (IECs) showed a decreased antimicrobial function in Paneth cells, with loss of their characteristic granules, a significant reduction in goblet cells, increased epithelial apoptosis and the development of intestinal enteritis via IRE1/XBP1 signaling, which was reversible under germ-free conditions (39, 82). Adolph et al. showed that the development of intestinal inflammation is promoted by stressed Paneth cells, as specific deletion of XBP1 in Paneth cells is sufficient to induce small intestinal enteritis (83). A study provided first evidence that intestinal ischemia/reperfusion induces UPR activation in the human small intestine, particularly in Paneth cells, and demonstrated subsequent induction of apoptosis in Paneth cells (84). Here, ER stress-induced Paneth cell apoptosis was shown to contribute to intesinal ischemia/reperfusion-induced bacterial translocation and systemic inflammation. With regard to epithelial **stem cells**, the UPR causes loss of self-renewal capacity in cells with ER stress (85, 86). Heijmans et al. showed that an activated UPR in crypt base columnar cells antagonizes stem cell properties and proliferation via stem cell-specific depletion of the ER chaperone Grp78 (87). More recently, they were able to show that heterozygosity of Grp78 in the intestinal epithelium compromises epithelial regeneration capacity and protects against adenoma formation (88). A similar mechanism to the UPR has been described in mitochondria and is termed the mitochondrial UPR (mtUPR) (89–91). Our lab showed that loss of the mitochondrial chaperone HSP60 activates the mtUPR resulting in mitochondrial dysfunction (92). In these mice, HSP60-deficiency causes a loss of stemness and cell proliferation in intestinal crypts. HSP60 deficiency in IECs triggered the paracrine release of Wnt-related signals associated with hyperproliferation of residual stem cells that escaped *Hsp60* deletion, demonstrating a fundamental role of mitochondrial function in the control of intestinal stem cell homeostasis. Under conditions of chronic inflammation, where this homeostasis is constantly challenged, this mechanism may contribute to inflammation associated tumorigenesis. In a recent study using mice in which Apc (Adenomatous polyposis coli; the most frequent initial gene mutation in CRC) and the ER stress chaperone Grp78 were deleted in IECs, it could be shown that ER stress signaling results in a rapid loss of Apc mutated stem cells and self-renewal capacity through interfering with Wnt signaling (93). The ER UPR and its role in intestinal pathologies will be one focus of this review, and is discussed in detail in subsequent sections.

Taken together, it has become clear that ER stress and the UPR signaling pathways play a pivotal role in shaping immune cell development and responses in order to mount an adequate immune response. Furthermore, IEC secretory cell function and the UPR play an important role in the maintenance of homeostasis and the resolution of inflammatory conditions. It is therefore not surprising that the UPR with its associated inflammatory pathways is also a key player in pathologies, including complex inflammatory, autoimmune, and neoplastic diseases.

## UPR and Intestinal Disease

ER stress and UPR activation critically impact the regulation of intestinal epithelial stem cell differentiation (87, 92), the development of chronic intestinal inflammation (39, 94, 95), and the pathogenesis of intestinal tumorigenesis (35). Understandably, a dysfunction of IECs, particularly highly secretory cells such as goblet cells, associates ER stress and the UPR with numerous gastrointestinal disorders such as inflammatory bowel disease (IBD), celiac disease, as well as cancer, including colorectal cancer (CRC) (96–98). With its multifaceted possibilities of physiological outcomes, understanding the role of the UPR in IBD and CRC will open up new avenues for treatments of these debilitating and life-threatening diseases.

### UPR in IBD

IBD refers to a group of multifactorial, immunologically-mediated chronic inflammatory diseases, of which Crohn's disease (CD) and ulcerative colitis (UC) represent the two major forms of disease. IBD can be debilitating and may lead to life-threatening complications, and its incidence and prevalence are increasing worldwide. The onset of IBD is suggested to result from genetic susceptibility, immune dysregulation, the intestinal microbiota, and environmental factors such as diet (99–102). Genome wide association studies (GWAS) over the past decade have identified numerous susceptibility loci for CD and UC, the latest of which links 241 susceptibility loci to IBD (103). Many of these susceptibility loci encode proteins with important roles in proteostasis. Among the ER-relevant genes identified here are Orosmucoid-like 3 (ORMDL3) (104, 105), anterior gradient 2 (AGR2) (106), and XBP1 (39). ORMDL3 has long been recognized as a key UPR inducer by disturbing endoplasmic calcium homeostasis (107). ORMDL3 was also shown to selectively activate the ATF6 arm of the UPR in lung epithelia (108, 109). The precise mechanism by which genetic abnormalities in ORMDL3 contribute to IBD is to date not understood, but it was shown to protect against apoptosis (110). The protein disulfide isomerase AGR2 is highly expressed in secretory cells, and mice deficient in AGR2 develop terminal ileitis and colitis displaying Paneth cell hypertrophy and a loss of mucin-filled goblet cells along with UPR activation (Grp78 increase) (56, 111). Similar to AGR2 deficiency, XBP1 deletion in IECs particularly affects secretory cells, with a loss of Paneth cells and mucin-filled goblet cells (39). IEC-specific XBP1 deletion resulted in ER stress (IRE1-XBP1 axis), spontaneous inflammation and an increased susceptibility to dextran-sodium sulfate (DSS)-induced colitis (39). *ATG16L1*^*T*300*A*^ is a major risk polymorphism in CD (112). Abnormalities in the secretory pathway of Paneth cells are a consequence of hypomorphic ATG16L1 (83, 113), and an IEC-specific deletion in mice *(Atg16l1*^Δ*IEC*^*)* demonstrates that the observed spontaneous transmural ileitis is driven by IRE1α, which accumulates in Paneth cells (114).

UC is characterized by depleted goblet cells and a reduced mucus layer (115, 116). Further evidence for a role of the UPR in goblet cells was provided by two strains of mice with distinct, non-complementing missense mutations in the major secreted intestinal mucin Muc2 (Winnie and Eeyore mice), which develop an UC-like phenotype (117). Goblet cells in these mice display evidence of ER stress and activation of the UPR, associating mucin misfolding and ER stress with the initiation of colitis in mice. Cytokines can either exacerbate or suppress ER stress and protein production in secretory cells. For example, IL-10 can act directly on goblet cells in the colon to reduce protein misfolding and ER stress and promote mucus barrier function (118). Furthermore, IL-22 was identified as a suppressor of ER stress, and was shown to reverse high-fat diet-induced intestinal epithelial stress and loss of mucosal barrier integrity (119, 120). Immunoglobulin A (IgA) is the major secreted immunoglobulin isotype found at mucosal surfaces. As discussed in the previous section, studies in mice with an IEC-specific deletion in XBP1 have shown that IEC-associated ER stress can serve as a nidus for spontaneous microbiota-dependent ileitis (39, 83). It was recently shown that IEC-associated ER stress induces the expansion and activation of peritoneal B1b cells, resulting in increased lamina propria and luminal IgA to induce a barrier-protective T cell-independent IgA response (121). This mechanism presents a beneficial self-contained host-derived response that occurs independently of the microbiota and inflammation.

Numerous models with perturbations in the UPR pathways (ATF6, p58IPK, IRE-1, CHOP, OASIS, and S1P) do not show spontaneous phenotypes but display increased susceptibility to DSS-induced colitis (45, 94, 122–124). Treton et al. reported that the coordinated expression of all three branches of the UPR is impaired in UC patient mucosa, and that a defective integrated stress response in these mucosa samples led to reduced ATF4 and CHOP transcripts and protein levels (125). Their findings demonstrate that inappropriate ER stress renders UC mucosa highly susceptible to pathological changes in the microenvironment and may present an *in vivo* signature for the susceptibility of unaffected UC mucosa to inflammation. Similarly, we observed a downregulation of CHOP mRNA and protein expression in mouse models of T-cell-mediated and bacteria-driven colitis (45). A further study conducted in our own group provided evidence of UPR activation (increased Grp78 expression) in IECs from IBD patients and a mouse model of intestinal colitis (IL-10^−/−^) (95). The finding that IL-10-mediated p38 signaling inhibited TNF-induced recruitment of ATF6 to the Grp78 promoter provides a plausible explanation for colitis development in IL10^−/−^ mice. Traditionally, Grp78 is regarded as a luminal ER chaperone, however numerous studies have established that, under cell stress conditions and in specific cell types, it can be found in other locations including the cell surface and the cytosol (126–128). This extends the functions of Grp78 beyond its traditional protein folding and processing role, to affect cell growth and signaling. In addition to a role of the UPR in IBD, mtUPR has been implicated in disease pathogenesis. Our study using double-stranded-RNA-activated protein kinase (PKR) knockout mice demonstrated that the highly selective mtUPR pathway employs PKR to recruit signaling molecules associated with the disease-relevant UPR signaling cascade, namely eIF2α and transcription factor activator protein-1 (AP1/cJun) (129). The observed eIF2α phosphorylation and AP1/cJun activation were dependent on activities of the mitochondrial protease ClpP and the cytoplasmic kinase PKR. The induction of mtUPR and PKR expression could be observed in murine IECs as well as patients with IBD, indicating that PKR may link mitochondrial stress to intestinal inflammation.

Taken together, there is substantial evidence from mouse models, patient data and GWAS studies, which demonstrate a role of the UPR (and mtUPR) in intestinal inflammation.

### UPR in CRC

Several studies suggest a complex relationship between ER stress and tumorigenesis due to the multifaceted outcomes of UPR activation, either by promoting pro-oncogenic adaptation and cellular survival or by acquiring pro-apoptotic tumor suppression (130, 131). Both cell extrinsic [hypoxia (132, 133), nutrient deprivation (134, 135), and acidosis (136, 137)] and cell intrinsic [oncogene activation (138–142), loss of tumor suppressor genes (143), chromosomal abnormalities (144)] factors can influence the tumor environment and therefore UPR signaling, to either mount a tumor-survival response (facilitating tumorigenesis) or an anti-tumor response (suppressing tumorigenesis). Sublethal tolerable levels of UPR activation allow adaptation to cell stress and sustain mechanisms of tumor progression, mostly through links between the ER stress response and fundamental biological processes, such as autophagy and ER-mitochondrial crosstalk (145).

Colorectal cancer (CRC) is one of the leading causes of death in the western society, being ranked third most lethal neoplasia in the United States in both men and women (146). As a key regulator of all UPR pathways, GRP78 constitutes a major marker for UPR signaling, and its enhanced expression correlates with the growth, invasion, and metastasis of tumors (147). Elevated levels of GRP78 could be observed in CRC cell lines and CRC patient tissue, with inhibition of GRP78 evoking enhanced sensitivity of CRC cells to chemotherapeutic agents (148). The selective contribution of the IRE1 pathway to an anticancer immune response in mice was demonstrated in three independent mouse cancer models fed a low-protein diet (149). Interestingly, an *Xbp1*-deficient epithelium results in an over-activation of IRE1α which drives the regenerative intestinal stem cell (ISC) expansion upon pathological ER stress, but is not involved in homeostatic ISC regulation (150). IRE1 signaling was also shown to induce vascular endothelial growth factor-A (VEGF-A), IL-β, and IL-6 during the process of CRC angiogenesis (151). Similar to IRE1, activation of the PERK pathway has also been shown to play a vital role in CRC initiation, progression and angiogenesis (152, 153). With respect to the causal role of the UPR effector ATF6 in tumor biology, very little is known, although its downstream target gene Grp78 is frequently found to be overexpressed (154). A missense polymorphism in ATF6 is associated with susceptibility to hepatocellular carcinoma (155). ATF6 mRNA expression positively correlates with CRC primary tumors and the likelihood of metastasis and relapse (156, 157). ATF6 was recently proposed as a marker for early dysplastic changes both in ulcerative colitis (UC)-associated and non-UC-associated CRC (158). In our newly generated transgenic mouse model expressing the active form of ATF6 in IECs (nATF6^IEC^), we observed spontaneous colorectal tumorigenesis through the induction of intestinal dysbiosis and innate immune response in the absence of early inflammation (35). Using germ-free mice, we showed that ATF6-activated UPR in the epithelium requires the presence of intestinal microorganisms for tumor formation. Our analysis of CRC patients in The Cancer Genome Atlas dataset identified aberrant ATF6 as a clinically relevant UPR mediator (35). Furthermore, our clinical results identified approximately 11% of CRC patients from all tumor stages who overexpressed ATF6, and linked increased ATF6 levels in tumors of a subset of CRC patients with increased risk of post-operative disease relapse, supporting our hypothesis that ATF6 represents a novel and clinically relevant tumor risk gene defining a subgroup of CRC patients.

The above findings demonstrate a clear involvement of the UPR in the different stages of CRC pathogenesis, however, it remains largely unclear how ER stress and the UPR promote survival of cancer cells. Cancer cells that are undergoing ER stress can actively modulate immune cell function through transmissible ER stress. Induced ER stress in cancer cells was shown to cause the upregulation of UPR genes and pro-inflammatory cytokines in responder macrophages (159). The same group showed that cell-extrinsic effects of tumor ER stress imprint myeloid DCs and impair CD8+ T cell priming (160). Further evidence for the modulation of immune cells through ER stress was provided by Lee et al. who showed that ER stress in tumor-bearing mice accelerated cancer progression and the immunosuppressive capacity of myeloid-derived suppressor cells (MDSCs) (161). It seems to be the magnitude of ER stress, which defines whether an immunosuppressive or immunogenic response is mounted.

**Figure 2:**
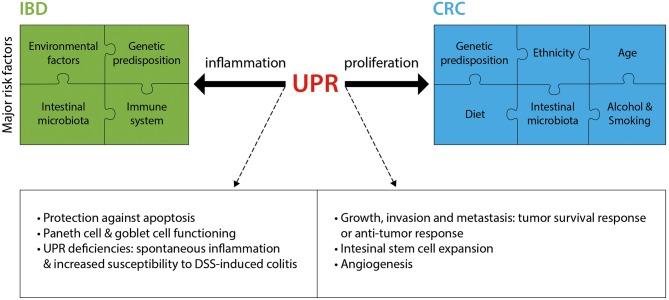
The UPR in IBD and CRC. IBD and CRC constitute complex diseases with numerous major risk factors contributing to disease pathology. The UPR significantly contributes to these two intestinal pathologies, mostly through an involvement in inflammation (IBD) and proliferation (CRC). Listed are the main known mechanisms by which the UPR is implicated in IBD and CRC. DSS, Dextran sodium sulfate.

As summarized in this section and in Figure 2, research over the past decade has provided much insight into the critical role played by the UPR in IBD and CRC. Fully elucidating the mechanisms by which the UPR promotes or prevents the progression of diseases such as IBD and CRC will pave the way for novel therapeutic approaches.

## UPR as a Pharmacological Target With Therapeutic Implications

As a key player in immunity and inflammation, the UPR has been increasingly recognized as an important pharmacological target, providing promising hope in the development of personalized therapeutic strategies for immune-mediated pathologies. To date there are several therapeutic opportunities involving the UPR. For descriptive and comparative purposes, these have been compiled in Table 2. Chemical chaperones are a group of low-molecular mass compounds that improve ER function. The most studied chemical chaperones are Tauro-ursodeoxycholic acid (TUDCA) and 4-phenyl butyrate (PBA) that, among other functions, were shown to reduce ER stress in intestinal epithelial cell lines (194), and in the intestinal epithelium where they decreased the severity to DSS-induced colitis in mice (162). In a further example, the clinically relevant Food and Drug Adnministration (FDA)-approved proteasome inhibitor Bortezomib sensitizes multiple myeloma cells to apoptosis through UPR induction (165). Increasing our molecular understanding of the intricate signaling pathways and their effects on the immune system is therefore indispensable to specifically and successfully target individual components of the UPR. Interestingly, a study by Wielenga et al. reported that ER stress-induced activation of the UPR forces colon cancer stem cells to differentiate, resulting in their enhanced sensitivity to chemotherapy *in vitro* and *in vivo* (195). These findings suggest that agents that induce the activation of the UPR may be used to specifically increase sensitivity of colon cancer stem cells to the effects of conventional chemotherapy. In light of the multitude of possible functions, responses and effects of the UPR, the therapeutic implications for inflammatory disorders and antitumor strategies in cancer may well be limitless. At the same time, however, it is indispensable to consider possible side effects when targeting the UPR, particularly with broad targeting approaches. This becomes evident with examples such as the receptor tyrosine kinase inhibitor sunitinib, which is FDA-approved for the treatment of renal cell carcinoma and gastrointestinal stromal tumors, but has also been shown to have negative effects on the anti-viral immune response (167).

**Table 2:** The UPR as a pharmacological target with therapeutic implications.

**Pharmacological agent**	**Function**	**Effect**	**References**
TUDCA	Small-molecule chaperone involved in ER protein folding	Reduces ER stress and restores glucose homeostasis in a mouse model of type II diabetes; reduces protein misfolding and colitis in mice	(162, 163)
PBA	Small-molecule chaperone involved in ER protein folding	Reduces ER stress and restores glucose homeostasis in a mouse model of type II diabetes; reduces protein misfolding and colitis in mice; alleviates LPS-induced lung inflammation	(162–164)
Bortezomib	26s proteasome inhibitor	Activates the PERK pathway to induce ATF4 and CHOP, and sensitizing multiple myeloma cells to apoptosis	(165)
Sunitinib	Receptor tyrosine kinase inhibitor	Affects tumor angiogenesis and tumor proliferation; influences IRE1α kinase activity and eIF2α phosphorylation; negative effects on anti-viral immune response	(166, 167)
(1) STF-083010 (2) MKC3946 (3) B-109 (4) MKC8866 (5) KIRA6 (6) KIRA8	IRE1α inhibitors	(1-4) tumor growth inhibition (1-2) increased apoptosis (4) increased survival (5) preservation of photoreceptors; improved glucose tolerance (6) β-cell protection; increased insulin secretion; prevents lung weight increase in lung fibrosis	(137, 168–175)
GSK2656157 GSK2606414	PERK inhibitors	Tumor growth inhibition; neuroprotection; increased glucose-stimulated insulin secretion	(176–183)
Compound 147	ATF6 activator	Reduced infarct size; preserved cardiac, kidney and neurological function; reduced liver triglyceride content	(184)
(1) Salubrinal (2) Guanabenz (3) Sephin 1	eIF2a phosphatase inhibitors	(1) Neuroprotection; positively and negatively affects survival (2) Neuroprotection; affects survival and disease onset; decreased axonal degeneration (3) Neuroprotection and motor recovery	(185–193)

## Concluding Remarks

Although our knowledge of the impact of ER stress and the UPR on immune responses requires much more insight and understanding, a growing body of new studies recognize the UPR as a fundamental mediator in cellular physiology and therefore also in the pathogenesis of inflammatory disorders, autoimmune responses, metabolic diseases, and tumorigenesis. Through its regulation of numerous cell-specific functions, the UPR is associated not only with the restoration of homeostasis, but also causally contributes to pathological processes. UPR signaling induces inflammatory responses, as well as inducing and controlling immune cell functions, making it an attractive research target with therapeutic implications for chronic immune-mediated diseases.

## Author Contributions

All authors listed have made a substantial, direct and intellectual contribution to the work, and approved it for publication.

### Conflict of Interest

The authors declare that the research was conducted in the absence of any commercial or financial relationships that could be construed as a potential conflict of interest.
